# Prognostic features and markers for testicular cancer management

**DOI:** 10.4103/0970-1591.60450

**Published:** 2010

**Authors:** Eddy S. Leman, Mark L. Gonzalgo

**Affiliations:** Department of Urology, Stanford University School of Medicine, Stanford, CA 94305, USA

**Keywords:** Non-seminoma, prognostic factors, seminoma, testicular cancer, tumor markers

## Abstract

Testicular neoplasm accounts for about 1% of all cancers in men. Over the last 40 years, the incidence of testicular cancer has increased in northern European male populations for unknown reasons. When diagnosed at early stage, testicular cancer is usually curable with a high survival rate. In the past three decades, successful multidisciplinary approaches for the management of testicular cancer have significantly increased patient survival rates. Utilization of tumor markers and accurate prognostic classification has also contributed to successful therapy. In this article, we highlight the most commonly used tumor markers and several potential “novel” markers for testicular cancer as part of the ongoing effort in biomarker research and discovery. In addition, this article also identifies several key prognostic features that have been demonstrated to play a role in predicting relapse. These features include tumor size, rete testis invasion, lymphovascular invasion, and tumor histology. Together with tumor markers, these prognostic factors should be taken into account for risk-adapted management of testicular cancer.

## INTRODUCTION

Testicular germ cell neoplasm represents approximately 1% of male cancers and are accountable for approximately 0.1% of cancer-related mortality in men.[1] In 2009, the American Cancer Society estimated that there were 8,400 new cases and 380 related deaths from testicular cancer.[1] Histologically, testicular germ cell cancer is divided into two major subgroups: seminoma and non-seminomatous germ cell tumors. Seminomas account for approximately 50% of testicular cancer and they arise most frequently in the fourth decade of life, whereas non-seminomatous germ cell tumors comprise 40% of testicular cancers and occur most frequently in the third decade of life. The remaining 10% of testicular cancers are combined tumors and they typically contain both seminomatous and non-seminomatous (embryonal cell carcinoma, yolk sac tumor, choriocarcinoma, and teratoma) elements. Understanding the disparity between seminoma and non-seminoma germ cell tumors is essential for the purpose of treatment and prognostication.

Several risk factors associated with the development of testicular germ cell cancers have been reported. These include prior history of germ cell tumor, cryptorchidism, testicular dysgenesis, and Klinefelter's syndrome.[2] A number of environmental factors such as maternal smoking during pregnancy, body mass index, and diet have also been reported to be associated with an increased risk of testicular cancer.[3] Increased levels of estrogen *in utero* and exposure of pregnant women to the nonsteroidal estrogen diethylstilboestrol (DES) have also been suggested as a potential risk factor.[4] However, studies by Dieckmann et al. have not confirmed the role of estrogen in the development of testicular germ cell cancer.[5] A study by Powel et al. suggests that HIV positive men are associated with increased risk of seminoma.[6] Gene analysis studies have also revealed that linkage to the Xq27 locus[7] and duplication or amplification of the short arm of chromosome 12 are also associated with the development of testicular cancer.[8] Although there has been no direct link between these factors and the risk of testicular cancer to date, these risk factors are continuously being evaluated for their roles in the development of the disease.

Over the last 40 years, the incidence of testicular cancer has doubled in Europe for unknown reasons.[9] Testicular cancer appears to be most common in northern European countries, with age-adjusted incidence rates between 4 and 10 in 100,000 men.[10] Nevertheless, the incidence rates are much lower in Asian, African, and African American men, where they range between 0.2 and 1 per 100,000 men.[10] The peak incidence of testicular cancer is typically between 15 to 35 years of age. When diagnosed at early stage, testicular cancer is typically curable with a high survival rate. Over the past 30 years, successful multidisciplinary approaches for the management of this disease have increased five-year survival rates significantly from approximately 63% to more than 90%.[10] In addition, utilization of both diagnostic and prognostic tumor markers, as well as accurate prognostic classification has contributed to highly effective therapy. This article reviews the utilization and discovery of tumor markers as well as application of different prognostic factors for testicular cancer that could potentially improve disease management.

## TUMOR MARKERS

Research efforts are ongoing to identify specific biomarkers (tissue and/or serum) that can improve diagnosis, surveillance of tumor progression/recurrence, and therapeutic response. To date, there are three relatively specific and sensitive serum biomarkers that are being used in the diagnosis, prognosis, and surveillance of testicular cancer. These serum markers include α-fetoprotein (AFP), human chorionic gonadothropin (HCG), and lactase dehydrogenase (LDH). Patients with elevated serum LDH, AFP, or HCG and their pre-chemotherapy levels have been integrated into the International Germ Cell Cancer Consensus Group (IGCCCG) consensus prognostic index for non-seminomatous classification. These patients are stratified into good, intermediate, and poor prognosis categories based on the primary tumor site, serum tumor marker levels, and whether extra pulmonary visceral metastases are present.[11]

AFP is a 70 kD glycoprotein produced by the fetal yolk sac, the liver, and the gastrointestinal tract. Increased levels of AFP are typically found in non-seminomatous tumors (embryonal carcinoma and yolk sac). The approximate half-life of AFP is 5–7 days.[12] AFP levels are typically not elevated in seminomas; however, if increased levels of AFP are found in pure seminoma, it must be considered and treated as a non-seminomatous germ cell tumor. Elevated serum HCG levels are typically present in both seminomas and non-seminomas. Increased levels of serum HCG following orchiectomy is an indication of persistent disease, whereas recurrence of HCG following chemotherapy- induced complete remission of metastatic disease indicates the presence of relapse. LDH is a less specific marker but has an independent prognostic value in men with advanced testicular cancer. It has been reported that LDH reflects the growth rate and tumor burden [12]. Increased levels of serum LDH have been reported in approximately 80% of advanced seminomas and in about 60% of non-seminomas.[12]

Although AFP, HCG and LDH are the most commonly used serum markers for management of testicular germ cell cancer, these markers are not very specific and they are only detected in approximately 60% of men with testicular cancer.[13] In addition, the sensitivity of these markers is limited, and the levels of these markers are usually “normal” in about 40% of men with disease recurrence.[13] In recent years, newly discovered biomarkers have been reported to differentiate carcinoma *in situ*, seminoma, embryonal carcinoma, teratoma and yolk sac tumor. Although it is beyond the scope of this review to discuss these markers extensively, we will highlight several “new” markers that have been reported in the literature. For instance, high mobility group proteins HMGA1 and HMGA2 are nuclear proteins that are expressed differently with respect to the state of differentiation of the testicular germ cell tumor.[14] Over-expression of HMGA1/2 has been reported in pluoripotent embryonal carcinoma, whereas loss of HMGA1 expression has been reported in yolk sac tumor, and that loss of HMGA1/2 expression has been shown in mature adult tissue of teratoma areas. Thus, different expression profiles of HMGA1/2 protein could be utilized as a tumor marker in testicular cancer cases with a problematic histological differential diagnosis.[14]

OCT3/4 is another marker that has been reported in testicular cancer. OCT3/4 is a transcription factor of the family of octamer-binding proteins (also known as the POU homeodomain proteins) and is considered as one of the key regulators of pluoripotency.[15] OCT3/4 has been reported as a well characterized marker for primodial germ cells[3] and its expression has also been reported in carcinoma in situ, seminoma, and embryonal carcinoma.[16–17] Although OCT3/4 could potentially be used as a marker for testicular cancer, various reports have shown that this marker is also expressed in normal adult stem cells and non-germ cell-derived cancers.[3] Therefore, more studies are clearly needed in order to address the specificity and sensitivity of this marker.

In addition to HMGA1/2 and OCT3/4, SOX proteins have also been reported as potential “new” markers for testicular cancer. SOX2 is a member of the SOX protein family and it is a transcription factor that regulates development and differentiation.[16–17] SOX2 expression has been reported in embryonal carcinomas, the undifferentiated part of non- seminomas, but it is absent in seminomas, yolk sac tumors and normal spermatogenesis.[18–19] Another SOX protein, SOX17 has also been demonstrated to discriminate carcinoma in situ and seminoma from embryonal carcinoma.[3] Although these SOX proteins show great potential as new biomarkers for testicular cancer, their suitability as functional diagnostic/ prognostic markers remain to be proven.

Through proteomic analysis, a set of nuclear structural proteins have been identified that are specific for seminomas.[20] Mass spectrometric and immunoblot analyses of these proteins revealed that one of the proteins identified in seminoma tissues appears to be CDK10 (Cell division protein kinase 10).[20] CDK10 is potentially involved in cell differentiation and growth, and thus may serve as a target for prognostication of seminomas. Although this is the first study to examine the role of nuclear structural proteins as potential biomarkers in testicular cancer, additional studies are clearly warranted to further identify the role of CDK10 as a potential tumor marker for testicular cancer. Other proteins that are involved in cell cycle regulation have also been utilized as potential prognostic markers for testicular cancer. In an immunohistochemical analysis of 19 seminoma and 64-non seminoma tissue samples, Pestacides et al. examined seven markers that were involved in cell cycle regulation.[21] The authors reported that among the markers examined, p53 and MIB were the only two markers that showed prognostic significance. p53 and MIB-1 at cut-off values of 10% and 30%, respectively, could predict the occurrence of progressive disease with approximately 50–60% sensitivity and 75-85% specificity.[21] Recently, cell-free circulating mitochondrial DNA in the sera of patients with testicular cancer has been shown to be a novel noninvasive biomarker for monitoring disease.[22] Although mitochondrial DNA levels were higher in men with testicular cancer, they did not correlate with any clinicopathological variables including pathological stage, lymph node invasion, and clinical stage.[22] Therefore, ongoing discovery and validation of testicular cancer biomarker(s) is crucial for improving disease management.

## TUMOR SIZE AND RETE TESTIS INVASION

With early diagnosis, clinical stage I seminoma has an excellent prognosis regardless of treatment regimen and the relapse rates are generally lower than those with stage I non-seminomas.[23] However, there have been a few reports on disease relapse on men with stage I seminomas.[24–26] Prognostic factors for seminoma typically involve size of the primary tumor and rete of testis invasion. In a nationwide Danish study of surveillance in 261 men with stage I seminoma, Von der Maase et al.[25] show that the testicular tumor size had a significant role as a prognostic factor for predicting relapse. The authors showed that the four-year relapse-free survivals were 94%, 82% and 64% for tumors < 3 cm, between 3 and 6 cm, and ≥ 6 cm, respectively. Similar observations have also been reported by other groups. For instance, Warde et al.[26] identified size of primary tumor > 4 cm and rete testis invasion as significant prognostic factors for relapse in men with stage I seminoma managed with surveillance. Parker et al,[24] performed a univariate analysis on 150 men with stage I testicular seminoma and found that the risk of relapse was associated with tumor diameter >6 cm, tumor invasion of rete testis, and lymphatic or vascular invasion. In addition, age (≤ 33 years old), as well as tumor infiltrating lymphocyte count were also identified as additional prognostic features capable of predicting relapse in men with stage I testicular seminoma.[24]

Further, a recent study by Choo *et al*.[27] examined outcomes and patterns of relapse in 88 clinical stage I seminoma patients who were managed with surveillance after orchiectomy. Of the 88 men, 17 experienced relapse. Using a Cox proportional hazard model, the authors showed that the presence of rete of testis invasion was a statistically significant predictive factor for disease relapse (hazard ratio 3.5, p=0.03). Overall, in comparison with non-seminomas, there is less information about prognostic features for seminomas. This could be due to the fact that there is a lower event rate for this disease. Thus far, size of the primary tumor and rete testis invasion seem to be the most utilized features for predicting relapse in testicular seminoma.

## LYMPHOVASCULAR AND VASCULAR INVASION

Lymphovascular invasion of the primary tumor has been demonstrated to be the most consistent prognostic factor for stage I non-seminomatous germ cell tumor. As shown by Fossa *et al*.[28] in a study of 102 men with stage I non-seminoma, 22 of the 102 men experienced relapse within one year after orchiectomy (median follow-up time of five months). In this group of men, lymphovascular invasion was identified as the most significant risk factor predicting relapse (p=0.0007). In a separate study, lymphovascular invasion was also identified as a significant poor prognostic factor, where 62% of men with lymphatic invasion developed distant metastases.[29] This is further confirmed by Colls et al. where they demonstrated that 46% of men with vascular lymphatic invasion in their primary tumor experienced relapse.[30]

Vascular invasion has also been identified as a prognostic factor for stage I non-seminomatous germ cell tumor. In an evaluation of 88 stage I non-seminomatous tumor specimens,[31] multivariate analysis of these samples showed that 23 of 88 patients with vascular invasion of the primary tumor had a high risk of relapse (61%, 95% CI 55-67%). A separate study showed that surveillance of 105 men with stage I non-seminomas revealed that 27/105 (25.7%) men had disease relapse. All relapses in this group of men occurred within two years of orchiectomy and vascular invasion was identified as one of the significant predictors of relapse during surveillance. Further, in examining the records of 82 patients with stage I non-seminomas following radical orchiectomy,[32] 30 of 82 patients did not have vascular invasion in their primary tumor, whereas 52 of 82 men had vascular invasion. In the group of men who had vascular invasion, 24 of 52 (46%) experienced relapse, thus indicating that vessel invasion could be used as a prognostic factor in monitoring stage I non-seminomatous germ cell tumor. Overall, these studies and others [33-36] have demonstrated that vascular invasion of the primary tumor is the most consistent prognostic feature identified in the management of stage I non-seminomas. However, pathological interpretation has been reported to vary between venous, lymphatic, and lymphovascular invasion.[37]

## EMBRYONAL CARCINOMA HISTOLOGY

Predominantly embryonal carcinoma histology is another prognostic feature that is frequently associated with the rate of disease relapse in stage I non-seminomatous testicular germ cell tumor. In a study that followed 132 patients (median follow-up 38 months) between 1978 and 2000, the relapse rate was 24% and all occurred before 23 months with 87% diagnosed within the first year. The presence of lymphovascular invasion, embryonal and yolk sac tumor were examined as risk factors in all specimens. The study concluded that the presence of embryonal carcinoma component was the only significant risk factor that could determine disease relapse.[38] Another group that evaluated 10-year results of a surveillance study of clinical stage I non-seminomatous cancer showed that 25 of 85 men experienced relapse after a median disease-free interval of seven months. [39] All 25 patients had predominant embryonal carcinoma histology in their primary tumor which was significantly associated with disease relapse (p=0.008). Dunphy *et al*.[29] also showed that in a study of 93 men with stage I non-seminomatous and mixed germ cell cancer who were placed in a surveillance study following orchiectomy, 81 men had predominantly embryonal carcinoma component in their primary tumor. Of these 81 men, 35% developed metastases, whereas none of the men without an embryonal carcinoma feature developed metastases (p=0.05).

## OTHER PROGNOSTIC FACTORS

A number of studies have identified other prognostic features that may play a role in predicting disease relapse in the stage I non-seminomatous testicular germ cell tumor. A prospective study of surveillance in 373 men with stage I non-seminomatous testicular cancer from 16 UK and Norwegian cancer centers was conducted by Read *et al*.[40] This study was performed to determine relapse-free rates and to identify the histological criteria that could predict relapse. The authors reported that the two-year and five-year relapse-free rates after orchiectomy were 75% and 73%, respectively. The authors further identified that the presence of undifferentiated cells and the absence of yolk sac elements in the primary tumor were able to identify a group of men with a high risk of relapse.

A study by Alexandre *et al*.[31] identifies the presence of mature teratoma as a prognostic factor capable of predicting disease relapse. However, the authors showed that in 88 tumor specimens from men with stage I non-seminomas undergoing surveillance, patients who had the presence of mature teratoma alone had a low risk of disease relapse compared to men who had vascular invasion in their primary tumor. Nevertheless, the authors concluded that the presence of teratoma histology was independently correlated with relapse free survival and thus could be used a factor to predict a subgroup of men with a very low risk of relapse. Other groups have also identified different histopathologic adverse prognostic predictors of relapse for stage I non-seminomatous testicular germ cell tumor. These include spermatic cord involvement[41] and trans-scrotal violation.[39] Although these features could potentially add to the management of stage I non-seminomatous testicular cancer, more studies are clearly needed in order to assess the clinical significance of these factors.

## PROGNOSTIC FACTORS IN METASTATIC DISEASE

A risk classification system is commonly used to stratify advanced testicular cancer according to prognostic information [Table 1].[42] Good risk non-seminoma is disease without evidence of nonpulmonary visceral metastases and post-orchiectomy markers all of: AFP < 1,000 ng/mL, HCG < 5,000 iu/L, and LDH <1.5 × upper limit of normal. Intermediate risk non-seminoma is disease without evidence of nonpulmonary visceral metastases and post-orchiectomy markers any of: AFP=1,000–10,000 ng/mL, HCG=5,000-50,000 iu/L, and LDH=1.5–10 × upper limit of normal. Poor risk non-seminoma is disease with a mediastinal primary tumor or nonpulmonary visceral metastases or post-orchiectomy markers any of: AFP > 10,000 ng/mL, HCG > 50,000 iu/L, and LDH > 10 × upper limit of normal.

**Table 1:** Risk classification of metastatic testicular cancer[42]

Risk Status	Nonseminoma	Seminoma

Good Risk	Testicular or retroperitoneal primary tumor and No nonpulmonary visceral metastasis and Post-orchiectomy markers all of: AFP < 1,000 ng/mL hCG < 5,000 iu/L LDH 1.5 × upper limit of normal	Any primary site and No nonpulmonary visceral metastases and Normal AFP Any hCG Any LDH
Intermediate Risk	Testicular or retroperitoneal primary tumor and No nonpulmonary visceral metastasis And Post-orchiectomy markers any of: AFP 1,00 – 10,000 ng/mL hCG = 5,000 – 50,000 iu/L LDH = 1.5 – 10 × upper limit of normal	Any primary site and Nonpulmonary visceral metastases and Normal AFP Any hCG Any LDH
Poor Risk	Mediastinal primary tumor or Nonpulmonary visceral metastasis or Post-orchiectomy markers any of: AFP > 10,000 ng/mL hCG > 50,000 iu/L LDH > 10 × upper limit of normal	No poor risk classification for seminoma

Metastatic seminoma is stratified into good and intermediate risk groups based on stage and tumor markers. Good risk seminoma is disease at any primary site without nonpulmonary visceral metastases and normal AFP, any HCG, and any LDH. Intermediate risk seminoma is comprised of disease at any primary site with nonpulmonary visceral metastases and normal AFP, any HCG, and any LDH. There is no poor risk classification group for seminoma.

## CONCLUSIONS

In this article, we reviewed the utilization of tumor markers and prognostic factors that are essential for the management of testicular germ cell tumors. Currently used and potential “novel” markers, as well as pathological features for prognostication of testicular cancer are summarized in Table 2. Discovery and validation of additional markers in combination with current markers (AFP, HCG, LDH) may facilitate improved diagnosis and disease management. A literature review of prognostic factors for seminomas reveals that primary tumor size and rete testis invasion is widely used for predicting relapse. In non-seminomas, vascular/ lymphovascular invasion, the presence of predominantly embryonal carcinoma histology and the absence of yolk sac tumor are commonly utilized prognostic factors for the disease. Taken together, these factors are clearly important for treatment and surveillance of testicular cancer.

**Table 2:** Tumor markers and prognostic factors for testicular cancer

	Seminoma	Non-seminoma

Currently available serum markers Potential “novel” markers	LDH,AFP, HCG OCT3/4 SOX2 SOX17 CDK10 Circulating mitochondrial DNA p53 and MIB-1	LDH,AFP, HCG HMGA1/2 (Embryonal carcinoma and teratoma) HMGA1 (Yolk sac tumor) OCT3/4 (Embryonal carcinoma) SOX2 (Embryonal carcinoma and yolk sac tumor) SOX17 (Embryonal carcinoma) Circulating mitochondrial DNA p53 and MIB-1
Commonly used histopathological features	Tumor size Rete testis invasion	Vascular/lymphovascular invasion Predominantly embryonal carcinoma histology Absence of yolk sac tumor
Other features	Age (≤ 33 years) Tumor infiltrating lymphocyte count	Spermatic cord involvement Trans-scrotal violation
